# Nano-therapeutics targeting the macrophage-based microenvironment in the treatment of atherosclerosis

**DOI:** 10.1186/s12967-025-07222-7

**Published:** 2025-10-24

**Authors:** Yingjie Xu, Yuan Zhang, Wanpeng Yu

**Affiliations:** 1 Qingdao Stomatological Hospital Affiliated to Qingdao University, Qingdao University, Qingdao, China; 2 Qingdao Medical College of Qingdao University, Qingdao University, Ningde Road 1, Qingdao, 266073 China; 3 Institute for Translational Medicine, The Affiliated Hospital of Qingdao University, Qingdao University, Qingdao, China

**Keywords:** Atherosclerosis, Macrophages, Microenvironment, Nanoparticles

## Abstract

Atherosclerosis (AS) is a chronic inflammatory disease that primarily affects large and medium-sized arteries and serves as the major pathological basis for cardiovascular diseases such as coronary heart disease. During the progression of AS, macrophages play a crucial role in promoting inflammatory regulation. Sustained local inflammatory responses are triggered by the accumulation of macrophages in arterial walls, which either promote or inhibit the development of AS by modulating inflammatory progression, plaque stability, and the surrounding immune microenvironment. Therefore, therapeutic strategies targeting macrophages and eliminating pro-inflammatory features in the plaque microenvironment hold promise as novel approaches to slow the progression of AS. With the deepening understanding of the mechanisms underlying AS, numerous innovative nanotherapeutic systems for its diagnosis and treatment have been developed. Here, we review strategies for designing novel nanosystems to treat AS, including modifying targeting ligands and utilizing biomimetic nanoparticles to enhance drug accumulation in target lesions and improve bioavailability. Macrophage-targeted nanotherapeutic approaches aim to reduce plaque burden and inflammation by regulating macrophage apoptosis, autophagy, and inducing efferocytosis synergistically. Concurrently, the development of intelligent responsive nanoparticles based on the inflammatory microenvironment enables targeted elimination of inflammatory characteristics within plaque microenvironments. These strategies demonstrate significant potential for application in AS treatment.

## Introduction

Atherosclerosis (AS) is a chronic multifocal inflammatory disease that typically occurs in medium and large arteries, characterized by fatty streak formation, fibrous plaques, and calcification. AS is the primary pathological basis of ischemic cardiovascular diseases such as coronary heart disease and cerebrovascular disease [1]. Extensive basic and clinical studies have demonstrated that AS has multiple risk factors, including hyperlipidemia, diabetes, and smoking. It is generally recognized as a vascular chronic inflammation triggered by the interaction of these risk factors with arterial wall cells. The earliest pathological event in the arterial intima is the adhesion of blood monocytes to endothelial cells. These monocytes then migrate into the subintimal layer, where they differentiate into macrophages. These macrophages ingest lipids such as oxidized low-density lipoprotein (ox-LDL) and other atherogenic lipoproteins, forming foam cells and lipid streaks, which constitute the main features of early-stage AS. Persistent inflammatory states at early lesion sites accelerate plaque progression, driving AS toward advanced stages. Late-stage lesions are marked by necrotic core formation and fibrous cap development, where the necrotic core is covered by the fibrous cap. The size of the necrotic core correlates with both the quantity of apoptotic macrophages and macrophage-mediated efferocytosis. These two hallmark features of late-stage lesions contribute to inflammatory microenvironments, oxidative stress (OS), thrombus formation, and promotion of adjacent cell death, thereby increasing plaque instability [2]. Ultimately, plaque rupture or endothelial erosion induces thrombosis, leading to clinical complications. Consequently, endothelial cells, macrophages, and intimal smooth muscle cells emerge as key players in disease progression [3]. The instability of advanced plaques may precipitate acute cardiovascular events, posing significant threats to patient survival. Persistent inflammation within atherosclerotic plaques (APs) is a critical factor contributing to plaque vulnerability and rupture. The pro-inflammatory microenvironment of plaques, characterized by increased monocyte recruitment, OS, and impaired clearance of apoptotic cells, plays a pivotal role in sustaining inflammation and hindering its resolution. Moreover, macrophages are central to the pathophysiology of many cardiovascular diseases, including AS. Chronic localized inflammatory responses arise from the accumulation of macrophages in the arterial wall, which triggers the release of chemokines, cytokines, and enzymes that degrade matrix proteins [4].

A hallmark pathogenic feature of AS is the accumulation of macrophages within the arterial wall [5]. Additionally, macrophages dynamically interact with vascular cells and drive phenotypic modulation of vascular smooth muscle cells [6]. As the primary source of enzymes that degrade matrix proteins, macrophages are pivotal to the rupture of APs. Therefore, therapeutic strategies targeting and eliminating pro-inflammatory features in the plaque microenvironment, particularly those focused on macrophages, hold promise as novel approaches to attenuate AS progression.

The most widely used therapeutic agents currently are small-molecule drugs, including statins, non-steroidal anti-inflammatory drugs (NSAIDs), and rapamycin. However, these drugs suffer from inherent limitations such as low water solubility, poor bioavailability, non-selective cellular uptake, and off-target deposition, which compromise therapeutic efficacy and risk damaging healthy tissues following systemic administration. Consequently, advanced drug delivery systems under development aim to overcome these challenges through more effective and selective delivery mechanisms.

Based on the growing understanding of AS mechanisms, numerous innovative nanotherapeutic systems for the diagnosis and treatment of AS have been continuously developed. Particularly in the research and development of bionanoparticles, various ligands have been designed to modify their surfaces, facilitating drug accumulation within target lesions and enhancing drug bioavailability. Utilizing macrophage-targeted nanotherapeutic strategies to reduce plaque burden and inflammation through synergistic effects of apoptosis, autophagy and induced efferocytosis in macrophages. Meanwhile, creating smart-responsive nanoparticles (NPs) based on inflammatory microenvironment to specifically target and eliminate inflammatory characteristics in plaque microenvironment. These strategies hold great potential for application in the treatment of AS.

## The mechanisms underlying the formation of the inflammatory microenvironment in AS 

In the microenvironment of APs, there are primarily cellular components (endothelial cells, macrophages, smooth muscle cells, etc.) and acellular components (LDL, pro-inflammatory cytokines, extravascular plasma concentration, etc.), along with various pathological factors that promote plaque formation within this microenvironment. Neovascularization participates in the accumulation of blood-derived components at atherosclerotic lesion sites, the formation of lipid cores and inflammatory microenvironments, and ultimately exacerbates plaque instability [7]. The chronic inflammatory microenvironment is a major factor driving the progression of AS. Current research has demonstrated that modulating inflammatory signaling pathways represents a potent therapeutic strategy for regulating AP progression, with regulation of reactive oxygen species (ROS) production being one of the effective therapeutic approaches. In normal tissues, there exists a balance between the production of ROS and the antioxidant components within the organism. However, at AP sites, due to the occurrence of OS, the levels of ROS generated in the plaque area increase significantly, with hydrogen peroxide (H_2_O_2_) being particularly abundant [8]. The accumulation of ROS promotes the oxidation of LDL to form ox-LDL, which is engulfed by macrophages to generate pathological foam cells. Excessive ROS accumulation also induces vascular cell damage, recruitment of inflammatory cells, lipid peroxidation, activation of metalloproteinases, and deposition of extracellular matrix. These processes collectively drive the progression of AS.

Moreover, inflammatory tissues are typically characterized by a mildly acidic microenvironment. In atherosclerotic lesions, the progressive development of plaques leads to persistent thickening of the vascular intima. When the distance between the deep intimal layer and the luminal surface exceeds the oxygen diffusion capacity threshold, localized hypoxia is induced. Under hypoxic conditions, electrons generated during glycolysis cannot utilize oxygen as the terminal electron acceptor, compelling the plaque tissue to shift to anaerobic glycolysis. This metabolic adaptation results in substantial lactate accumulation, consequently leading to a decline in local pH values and the establishment of a mildly acidic microenvironment [9]. The acidic environment significantly inhibits lysosomal enzyme activity (e.g., lysosomal acid lipase, LAL), impairing the degradation of ox-LDL. This dysfunction promotes lipid droplet deposition and accelerates foam cell formation. Furthermore, low pH activates apoptotic pathways through mitochondrial or death receptor mechanisms, releasing pro-inflammatory mediators. Notably, APs harbor multiple enzyme classes, including matrix metalloproteinases (MMPs), hyaluronidase (HAase), and cathepsins (Cts). These enzymes degrade extracellular matrix components, exacerbating local inflammatory responses and facilitating the development of thin fibrous caps—a hallmark of vulnerable plaques. Thus, the inflammatory microenvironment of vulnerable plaques is characterized by three cardinal features: acidic pH, excessive ROS, and elevated proteolytic enzyme activity [10].

During the progression of AS, macrophages play a pivotal role in mediating inflammatory regulation. As phagocytic cells derived from monocytes, macrophages exhibit phenotypic plasticity across functional stages and serve indispensable functions throughout the development of atherosclerotic lesions. As the predominant immune cell population within APs, macrophages exert dual regulatory functions through polarization into pro-inflammatory M1 and anti-inflammatory M2 phenotypes. These specialized cells participate in multiple pathological processes encompassing plaque formation, progression, rupture, and tissue repair. By dynamically influencing inflammatory cascades, plaque stability, and the peri-plaque immune microenvironment, macrophages serve as crucial determinants of atherosclerotic pathogenesis, either promoting or restraining disease development through these multifaceted mechanisms [11, 12]. M1 macrophages increase lipid accumulation, secrete pro-inflammatory factors, and promote foam cell formation; whereas M2 macrophages reduce lipid accumulation, release anti-inflammatory factors, and inhibit foam cell formation. The polarization phenotype of macrophages is influenced by the microenvironment of atherosclerotic plaques. Lipids and their derivatives, which are abundant in atherosclerotic plaque regions, can regulate the polarization of macrophages. Studies have shown that cholesterol crystals can induce macrophage polarization toward the M1 phenotype. M1 macrophages can activate the NLRP3 inflammasome, promote inflammatory progression, and release interleukin-1 (IL-1) and interleukin-8 (IL-8), thereby contributing to the formation of AS [13]. The continuous accumulation of ox-LDL in plaques can induce the transformation of M2 macrophages to the M1 phenotype, cause damage to vascular endothelial cells, and increase the secretion of pro-inflammatory cytokines such as interleukin-6 (IL-6), IL-8, and monocyte chemoattractant protein-1 (MCP-1). The increase in M1 macrophages, endothelial cell injury, and elevated secretion of pro-inflammatory factors are major drivers of atherosclerotic plaque rupture. Therefore, it is crucial to regulate the progression of AS by clearing ROS in plaque macrophages to alleviate OS damage and modulate macrophage polarization in the microenvironment. Studies have confirmed that the aggregation level and activation status of macrophages are positively correlated with the severity of atherosclerotic lesions. Regulating macrophage polarization can effectively manage and reduce foam cell formation, thereby opening new possibilities for the treatment of cardiovascular diseases such as AS. Furthermore, alleviating plaque burden and inflammation by modulating macrophage apoptosis, autophagy, and efferocytosis also represents an effective therapeutic strategy against AS. Depending on the stage of plaque development, macrophage apoptosis has varying effects on the progression of AS. In the early stages of the disease, macrophage apoptosis is considered beneficial as it reduces the cellular density of lesions and slows plaque formation. However, when increased macrophage apoptosis induced by pro-inflammatory factors leads to impaired phagocytic function, it tends to promote the formation of atherosclerotic plaques [14]. Therefore, the phagocytic function of macrophages plays a critical role during the development of AS. Understanding the mechanisms of macrophage death may provide a theoretical basis for preventing AS.

With the emergence of epigenomic and epigenetic modifications associated with AS risk, the etiology of AS has shifted from lipid disorders, inflammation, and metabolic dysregulation to epigenetic disease. Macrophage polarization is meticulously regulated by metabolites/metabolism and epigenetic modifications such as DNA methylation, histone modifications, and non-coding RNAs. Therefore, regulating macrophage polarization through epigenetic and metabolic modifications also represents a potential therapeutic strategy for AS [15]. Studies have shown that some “druggable” metabolic regulators (such as PFKFB3, PPARα, and AMPK) and regulators related to cholesterol transport (such as ABCA1, ABCG1, and SR-BI) are associated with the anti-inflammatory polarization of macrophages [16, 17]. Furthermore, research has found that certain drugs, including conventional Western medicines and traditional Chinese medicines, can modulate macrophage polarization. In-depth exploration of the specific mechanisms of these drugs may provide new insights for the prevention and treatment of AS [18, 19]. In other related diseases, regulators that modulate macrophage/monocyte infiltration and macrophage polarization, such as growth hormone secretagogue receptor (GHSR-1a) and Pellino E3 ubiquitin protein ligase 1 (Peli1), also play significant roles in the formation of atherosclerotic plaques [20–22]. Therefore, in the context of AS, identifying key regulators in the process of macrophage polarization is also crucial for discovering new targets for drug development.

## Macrophage-targeted nanotherapeutic strategies for AS

**Fig. 1:**
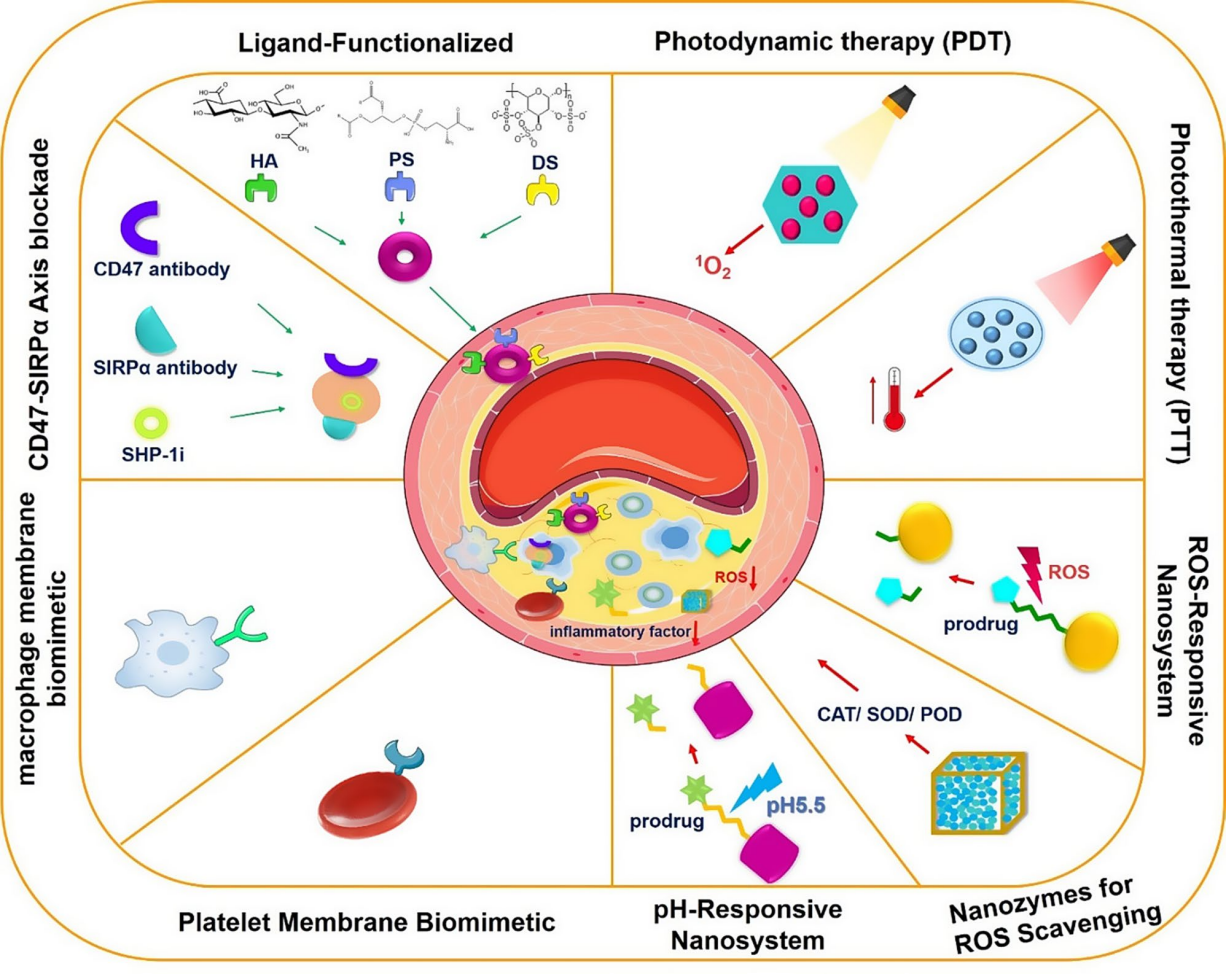
Schematic for nano-therapeutics targeting the macrophage-based microenvironment in the treatment of atherosclerosis

Given the pivotal role of macrophages in AS progression, reducing pro-inflammatory macrophages could attenuate inflammatory responses. Thus, modulating macrophage responses is recognized as a promising therapeutic approach for AS [23]. Currently, two macrophage-targeted strategies exist for AS treatment: macrophage ablation and functional reprogramming. Multiple nanoplatforms have been engineered to implement macrophage-targeted therapies, alleviating plaque burden and inflammation through coordinated regulation of macrophage apoptosis, autophagy, and efferocytosis induction (Fig. 1).

### Ligand-Functionalized macrophage-targeted systems

Macrophages play a critical role in AS, making them important targets for disease-related diagnosis and therapy, particularly due to their inherent ability to passively and actively uptake NPs. Passive targeting primarily involves the extravasation of nanomaterials from the bloodstream into atherosclerotic plaques, where macrophages accumulate, following systemic circulation. Owing to their phagocytic capability, macrophages can intrinsically internalize nano- or micro-sized particles. However, magnetic NPs with varying sizes, shapes, densities, and magnetic strengths can influence macrophage phagocytosis. For instance, NPs with high positive or negative surface charges are preferentially internalized by macrophages. Nevertheless, cationic or anionic NPs are prone to activate the complement system and are subsequently cleared from circulation. In contrast, neutral or zwitterionic particles exhibit prolonged circulation times [24, 25]. Active targeting, on the other hand, is achieved by modifying nanomaterials to introduce ligands that bind to specific receptors on the macrophage surface. Macrophages express a variety of receptors that play crucial roles in mediating phagocytosis, including glycoproteins, integrins, and Toll-like receptors, among others. Studies have shown that polysaccharides can bind to receptors on the macrophage surface, trigger signal transduction, activate the release of immune factors, and thereby modulate macrophage function. Additionally, polysaccharides can enhance the phagocytic activity of these cells. For example, CD36 and scavenger receptor A (SR-A) are expressed at low levels in normal arterial endothelial cells but are highly expressed in macrophages within plaque areas, where they mediate the uptake of ox-LDL by macrophages. Phosphatidylserine (PS) can specifically target the CD36 receptor on macrophages [26]. The negatively charged macromolecule Dextran Sulfate (DS) acts as a specific ligand for SR-A and selectively binds through ligand-receptor recognition to the positively charged SR-A residues on the surface of macrophages or foam cells in plaque regions. Mannose-modified NPs leverage the overexpression of mannose receptors (CD206) on polarized M2 macrophages to enhance cellular uptake. Hyaluronic acid-coated nanoparticles target the CD44 receptor on macrophages and improve drug delivery in models of inflammatory diseases. By modifying the surface of NPs with functional ligands that can specifically bind to overexpressed biomarkers within APs, these NPs achieve targeted accumulation in plaque areas. This modification enables them to evade clearance by the reticuloendothelial system (RES), thereby enhancing drug delivery to disease sites and improving drug bioavailability. Additionally, this approach facilitates macrophage-targeted drug delivery for AS treatment. Current common ligand modification strategies include functionalization with hyaluronic acid, phosphatidylserine, and dextran sulfate (Table 1).

**Table 1:** Macrophage-targeted nanotherapeutic strategies for atherosclerosis

	Nanoparticles	Targeting ligands	Reception	Carriers	Loaded drug	Models	Therapeutic effects	References
Ligands-modified	HA-CC-RST	Hyaluronic acid (HA)	CD44	hybrid liposomal cerasomes	Rosuvastatin (RST)	ApoE^−/−^ mice fed a high-fat diet	Reduce pro-inflammatory cytokine levels and foam cell formation; reduce plaque area and volume by 56.3% and 78.7%, respectively	[27]
	apoA-I/PS-NP^2^_S/P/C_	Phosphatidylserine (PS)	SR-BI/CD36	rHDL shell	Pitavastatin (PT); SR-A siRNA; catalase	ApoE^−/−^ mice fed a high-fat diet	Reduce intracellular lipid accumulation; reduce plaque areas by 65.8% and decrease macrophages by 57.3%; reduce plaque area by 66.5%	[28]
	DS-Ce6	Dextran sulfate (DS)	scavenger receptor-A (SR-A)	chlorin e6 (Ce6)	——	ApoE^−/−^ mice fed a high-fat diet	Reduce macrophage areas by 57.6%; reduce plaque area by 29.2%	[29]
CD47-SIRPα Axis-Modulating	aCD47@PMSN	CD47 antibody	CD47	mesoporous silica nanoparticle (PMSN)	——	ApoE^−/−^ mice fed a Western-type diet	Increase the phagocytosis to 79%; reduce plaque area by 16.6%	[34]
	ASOs@CaP-aSIPRα	SIRPα antibody	SIRPα	calcium phosphate (CaP)	anti-sense oligonucleotides of mammalian target of rapamycin (mTOR ASOs)	ApoE^−/−^ mice fed a high-fat diet	Enhance phagocytosis of apoptotic cells by macrophages; reduce plaque area by 10.3%	[35]
	SWNT-SHP1i	——	——	single-walled carbon nanotubes (SWNTs)	a chemical inhibitor of Src homology 2 domain-containing phosphatase-1 (SHP1i)	LDLR^−/−^ pigs fed a high fat/high calorie Western diet	Enhance phagocytosis; reduce plaque vulnerability and lesion size	[36]
	UM-EV_Lipo_	extracellular vesicles (EVs)	macrophage	urease nanomotors (UM)	SHP-1 siRNA	ApoE^−/−^ mice fed a high-fat diet	Promote plaque macrophage efferocytosis; significant clear the plaque in the aorta	[37]
	CEZP	——	——	metal − organic frameworks (MOFs)	CpG	ApoE^−/−^ mice fed a high-fat diet	Promote cholesterol efflux; ROS clearance; promote efferocytosis; significantly reduce total cholesterol (TC) and LDL levels; enhance plaque stability	[38]
Macrophage membrane-biomimetic modified	MM@Lips-SHP1i	macrophage membrane (MM)	macrophage	liposome NPs	SHP1i	ApoE^−/−^ mice fed a high-fat diet	Enhance efferocytosis; reduce plaque area by 67.49%	[40]
	MM/RAPNPs	MM	macrophage	poly(lactic-co-glycolic acid) (PLGA)	Rapamycin (RAP)	ApoE^−/−^ mice fed a high-fat diet	Reduce the average area of necrotic cores by 12.84%	[41]
	MM@DA-pCD@MTX	MM	macrophage	poly(isobutenealt-maleic anhydride) (PIAMA)	dopamine (DA)/methotrexate (MTX)	ApoE^−/−^ mice fed a high-fat diet	Reduce lipid deposition in plaques by 11.38%; reduce cholesterol deposition in the aorta by 5.25%; reduce ROS; TC, LDL-C, and TG levels were significantly reduced; the atherosclerotic plaque rates significantly lower to 2.49%	[42]
Platelet membrane-biomimetic modified	PEG-MPDA	Platelet membrane (PM)	macrophage	mesoporous polydopamine (MPDA)	probucol	ApoE^−/−^ mice fed a high-fat diet and performed partial carotid ligation surgery	Reduce ROS; inhibit LDL oxidation; reduce cell foaming; reduce the lesion area of plaques	[44]
	RAP@PLT	PM	macrophage	PLGA	RAP	ApoE^−/−^ mice fed a high-fat diet	Decrease lipid deposition and collagen levels; reduce plaque area by 19.25%; inhibit the proliferation of SMCs and macrophages in plaques	[45]
	PBP@siR@PM	PM	macrophage	black phosphorus nanosheets (BPNSs)	CaMKIIγ siRNA	ApoE^–/–^ mice fed a high-fat diet	Reduce ROS; attenuate oxidative stress and inflammatory response; stabilize the plaque; enhance efferocytosis;	[46]

#### Hyaluronic acid (HA)-Modified nanoparticles

Hyaluronic acid (HA) is the primary ligand for CD44, a multifunctional transmembrane glycoprotein overexpressed in activated macrophages. Therefore, active-targeting nanocarriers incorporating HA ligands specific to CD44 selectively accumulate in APs by targeting inflammatory macrophages within the lesions. For example, Ma et al. developed HA-modified hybrid liposomal cerasomes (CCs) loaded with rosuvastatin (RST), termed HA-CC-RST. The HA modification enabled HA-CC-RST to selectively target CD44-positive macrophages within plaques, thereby achieving precise delivery to atherosclerotic lesions. Results demonstrated that HA-CC-RST exhibited significantly higher local accumulation in CD44-overexpressing plaques compared to free RST, leading to marked plaque regression. In murine models, HA-CC-RST treatment reduced pro-inflammatory cytokine levels and foam cell formation while upregulating anti-inflammatory genes such as CCL5, CCR6, and CXCR5. More importantly, HA-CC-RST treatment reduced plaque area and volume by 56.3% and 78.7%, respectively, and significantly inhibited plaque progression. These findings highlight HA’s dual advantages of specific targeting and biocompatibility, making HA-modified nanocarriers a promising tool for both diagnosis and treatment of AS [27].

#### Phosphatidylserine (PS)-Modified nanoparticles

Phosphatidylserine (PS), abundantly present on apoptotic cell membranes, acts as a specific “eat me” signal during apoptosis. This signal is recognized by macrophages through high-affinity interactions with scavenger receptor class B type I (SR-BI) and CD36 receptors, triggering macrophage phagocytosis to exert anti-inflammatory effects. Leveraging this endogenous biomaterial, PS can serve as a platform for constructing targeted drug delivery systems. For instance, Jiang et al. designed a multifunctional dual-targeting biomimetic nanosystem comprising an ATP-responsive ternary polymeric core (for complexing SR-A siRNA and catalase) and a PS-modified, reconstituted high-density lipoprotein (rHDL)-based shell loaded with pitavastatin. This system dynamically enhanced macrophage targeting of CD36 in plaques through SR-A and CD36 co-regulation, significantly improving atherosclerotic plaque targeting efficiency and boosting macrophage cholesterol efflux capacity. After three months of treatment, plaque area and macrophage content in murine models were reduced by 65.8% and 57.3%, respectively [28].

#### Dextran sulfate (DS)-Modified nanoparticles

Dextran sulfate (DS), a ligand for scavenger receptor-A (SR-A), can be used to functionalize nanoparticles for targeting inflammatory macrophages. SR-A is overexpressed on activated macrophages—an early pathogenic alteration observed in atherosclerosis. Since SR-A overexpression occurs exclusively in activated macrophages, this mechanism can be leveraged for precise macrophage targeting. For example, Song et al. developed DS-modified nanoparticles conjugated with chlorin e6 (DS-Ce6 NPs) for photodynamic therapy (PDT) in AS. DS enables macrophage targeting via specific recognition of SR-A, a key receptor involved in modified LDL uptake and foam cell formation. Experimental results demonstrated that DS-Ce6 NPs induce macrophage autophagy, ultimately reducing plaque burden and inflammation. These findings highlight macrophage-targeted PDT as a potent strategy for treating APs [29].

### CD47-SIRPα axis-modulating nanotherapeutics

In recent years, CD47-SIRPα signaling pathway blockade therapy has garnered increasing attention for treating atherosclerosis. CD47 (cluster of differentiation 47), also known as integrin-associated protein (IAP), is a ubiquitously expressed transmembrane glycoprotein with a molecular weight of 50 kDa [30]. As the ligand for signal regulatory protein-α (SIRPα) on macrophages, CD47 inhibits macrophage-mediated efferocytosis of foam cells [31, 32]. Pathological upregulation of CD47 impairs efferocytic function, leading to necrotic core accumulation, thinning of the fibrous cap, and increased plaque rupture risk [33]. Blocking the CD47-SIRPα pathway represents a potential strategy to modulate the plaque immune microenvironment and suppress disease progression. CD47-SIRPα blockade has been shown to inhibit atherosclerotic plaque progression by restoring defective efferocytosis and enhancing foam cell clearance (Table 1).

CD47 antibody-functionalized nanoparticle platforms aim to treat AS by restoring macrophage efferocytosis. For instance, Chen et al. developed a platelet membrane-coated mesoporous silica nanoparticle (PMSN) system loaded with anti-CD47 antibodies, termed aCD47@PMSN. Leveraging the homing ability of platelet membranes, this nano-system selectively delivers CD47 antibodies to APs. By blocking CD47 on smooth muscle cells within the necrotic core, aCD47@PMSN promotes macrophage-mediated efferocytosis of necrotic cells, thereby clearing cellular debris and stabilizing plaques. In an apolipoprotein E knockout (ApoE^−/−^) mouse models of atherosclerosis, aCD47@PMSN significantly enhanced efferocytosis of necrotic cells in plaques. This clearance reduced AP area, stabilized plaque structure, and lowered risks of plaque rupture and late-stage thrombotic events [34].

In addition, NP platforms functionalized with SIRPα antibodies can restore macrophage autophagy to combat AS. For instance, Liu et al. designed a targeted nanosystem termed ASOs@CaP-aSIRPα, which integrates acid-responsive calcium phosphate (CaP) nanoparticles loaded with antisense oligonucleotides (ASOs) targeting the mammalian target of rapamycin (mTOR). These CaP NPs were coated with lipids (ASOs@CaP) and further modified with anti-SIRPα antibodies (aSIRPα). This system accumulates in APs, targets macrophages, and reactivates efferocytosis by blocking the CD47-SIRPα signaling axis. Concurrently, the mTOR ASOs suppress mTOR expression, restoring impaired autophagy to reduce apoptotic cell accumulation and lipid deposition. In murine AS models, ASOs@CaP-aSIRPα restored macrophage clearance of apoptotic cells, activated autophagy, and significantly reduced plaque area, inflammation, and endothelial dysfunction, thereby stabilizing plaques. This nanotherapeutic approach markedly attenuated plaque burden and suppressed atherosclerotic progression [35].

Furthermore, the nanoparticle system enables efficient delivery and enrichment of functional miRNAs and siRNA within APs. By utilizing SHP-1 siRNA to suppress the CD47-SIRPα signaling pathway — a key inhibitor of efferocytosis (the phagocytic clearance of apoptotic cells) — this approach promotes the removal of necrotic cells by macrophages, ultimately reducing plaque burden. Bamezai et al. developed a macrophage-specific nanotherapy based on single-walled carbon nanotubes (SWNTs) loaded with a chemical inhibitor of Src homology 2 domain-containing phosphatase 1 (SHP-1), termed SWNT-SHP1i. In LDLR^−/−^ pigs models, SWNT-SHP1i selectively delivered the drug to inflammatory monocytes and macrophages within APs, effectively enhancing phagocytosis and increasing the clearance of apoptotic cells. This approach significantly reduced plaque vulnerability and lesion size [36]. Zheng et al. constructed a biomimetic nanorobot (UM-EV_Lipo_) by hybridizing urease nanomotors (UM) with macrophage-derived extracellular vesicles (EVs) and SHP-1 siRNA-loaded liposomes. This system maximizes macrophage efferocytosis by reprogramming macrophage phenotypes and blocking the CD47-SIRPα signaling pathway. The urease nanomotors enhance endothelial barrier penetration, enabling UM-EV_Lipo_ to accumulate within APs, reactivate impaired efferocytosis, and reduce carotid and coronary plaque burden. In vivo and in vitro studies demonstrated that UM-EV_Lipo_ boosts macrophage phagocytic function in plaques, rapidly and precisely treating AS through necrotic tissue clearance and plaque regression [37]. Tang et al. developed a multifunctional nanotherapeutic system (CEZP) composed of a zinc ion (Zn²⁺) and epigallocatechin gallate (EGCG)-assembled metal-organic framework (MOF) encapsulating CpG oligodeoxynucleotides. The CEZP NPs synergistically combine three therapeutic mechanisms, including CpG enhances macrophage phagocytosis of apoptotic cells and foam cells with elevated CD47 expression; and Zn²⁺ promotes intracellular lipid degradation, while EGCG upregulates ATP-binding cassette (ABC) transporters to boost cholesterol efflux. Additionally, CEZP exhibits antioxidant and anti-inflammatory properties, driving the repolarization of pro-inflammatory M1 macrophages to anti-inflammatory M2 phenotypes. In murine AS models, intravenous administration of CEZP led to effective accumulation in APs, enhanced macrophage phagocytosis, and stabilization of plaque structure. CEZP significantly reduced plaque burden and modulated macrophage phenotypes through the coordinated actions of CpG, Zn²⁺, and EGCG, offering a novel combinatorial strategy for treating chronic AS [38].

### Macrophage membrane-biomimetic nanosystem

The unique components within the complex microenvironment of APs enable specific interactions with macrophages [10]. Macrophage membrane-coated nanoparticles are recognized as an excellent active-targeting platform for therapeutic drug delivery. The macrophage membrane coating not only protects NPs from immune clearance but also enables precise recognition of plaque-specific receptors via membrane-expressed ligands, such as P-selectin glycoprotein ligand-1 (PSGL-1), L-selectin, lymphocyte function-associated antigen 1 (LFA-1), integrin α4β1, and very late antigen-4 (VLA-4). These ligands mediate recruitment to APs by binding to their cognate receptors [39]. Thus, macrophage membrane-camouflaged NPs leverage the innate “homing” mechanism of macrophages to infiltrate atherosclerotic lesions, enabling targeted and effective treatment of AS (Table 1).

Functionalized macrophage membrane biomimetic nanoplatform exerts therapeutic effects through competitive macrophage inhibition, significantly attenuating foam cell generation and downregulating inflammatory mediators (cytokines/proteins). Additionally, the platform enhances macrophage lipid-handling capacity and autophagy function, accelerating cholesterol efflux to inhibit foam cell generation. It also promotes the phenotypic transition of pro-inflammatory M1 macrophages to anti-inflammatory M2 macrophages, thereby mitigating inflammatory damage. Furthermore, the nanoplatform induces apoptosis of foam cells and scavenges ROS, collectively contributing to its therapeutic efficacy against atherosclerotic progression.

For instance, Sha et al. developed macrophage membrane-coated biomimetic NPs (MM@Lips-SHP1i) by encapsulating lipid NPs loaded with SHP1i, a downstream small molecule inhibitor of CD47, within macrophage membranes. These biomimetic NPs retained inherent membrane proteins and functions of macrophages, enabling effective evasion of immune system clearance while actively targeting and accumulating in APs. Within plaque regions, MM@Lips-SHP1i NPs competitively bind with ox-LDL and lipopolysaccharide (LPS) in vivo, reducing macrophage uptake of new lipids. This dual mechanism decreases foam cell formation and suppresses pro-inflammatory cytokine expression. Furthermore, the SHP-1 small molecule inhibitor carried within the macrophage membrane biomimetic NPs blocks CD47-SIRPα signaling transduction in monocytes and macrophages. This enhances macrophage efferocytosis while inhibiting plaque progression, achieving synergistic therapeutic effects against AS [40]. Wang et al. developed macrophage membrane biomimetic NPs (MM/RAPNPs) by coating rapamycin (RAP)-loaded poly(lactic-co-glycolic acid) (PLGA) NPs (RAPNPs) with macrophage membranes (MM). These biomimetic NPs retained functional proteins, including specific integrin α4 and β1 signaling molecules, as well as CD47 proteins, thereby enhancing drug accumulation within AP regions and actively targeting dysfunctional endothelial cells. In vitro experiments demonstrated that MM/RAPNPs alleviated LPS-induced inflammation and inhibited the proliferation of macrophages and smooth muscle cells (SMCs). In mouse models, treatment with MM/RAPNPs reduced lipid content in plaques and significantly decreased necrotic areas [41]. Zhu et al. constructed biomimetic NPs (MM@DA-pCD@MTX) by modifying dopamine (DA) and methotrexate (MTX)-loaded amino-functionalized β-cyclodextrin (NH2-β-CD) onto the backbone of poly(isobutene-alt-maleic anhydride) (PIAMA), followed by coating with macrophage membranes. Leveraging the “homing” capability of functionalized macrophage membranes (MM), MM@DA-pCD@MTX enabled targeted drug delivery to APs while prolonging systemic circulation time and drug half-life. In vivo results demonstrated that the synergistic effect of β-cyclodextrin (β-CD) and methotrexate (MTX) upregulated the expression of ABCA1, CYP27A1, and SR-B1, enhanced cholesterol crystal solubility, and thereby promoted cholesterol efflux while suppressing foam cell formation. Additionally, dopamine (DA) within the nanoparticles scavenged excessive ROS, inhibited lipid peroxidation, and alleviated OS at plaque sites. This multifunctional nanoplatform thus represents a promising system for targeted AS therapy [42].

Macrophage membrane biomimetic nanomaterials exhibit characteristics such as low immunogenicity, prolonged circulation time, and strong targeting ability, which can enhance the treatment efficiency of AS. However, there are some limitations: (1) The in vitro conditions required to maintain the activity of functional surface proteins on macrophage membranes are relatively stringent, hindering their clinical application; (2) The immunogenicity between macrophage membrane donors and recipients after entering the human body may lead to membrane damage. Overall, macrophage membrane biomimetic NPs show promising prospects in the treatment of AS. With advances in nanomedicine research, functionalized macrophage membrane biomimetic NPs loaded with multiple drugs are expected to demonstrate even more promising applications in the comprehensive treatment of AS in the future.

### Platelet membrane-biomimetic nanosystem

Platelets play a pivotal role in the initiation and progression of AS. Under inflammatory conditions, the accelerated recruitment and aggregation of platelets contribute to the erosion or rupture of vulnerable plaques in late-stage thrombogenesis. The activation, aggregation, and ultimate prothrombotic functions of platelets involve interactions with blood-exposed endothelial cells and extracellular matrix components, indicating platelets’ inherent affinity for APs and their natural capacity for plaque recruitment [43]. Consequently, mimicking the intrinsic adhesive functions of platelets may represent an effective strategy for targeting plaques, which has significantly promoted their application in functionalized nanoplatforms. NPs coated with platelet membranes can interact with activated endothelial cells, foam cells, and collagen within plaques. This novel biomimetic strategy utilizing platelet membrane-coated NPs enables targeted drug delivery for the treatment of APs (Table 1).

Chen et al. leveraged the properties of platelet membranes to develop a mesoporous polydopamine (MPDA) NP loaded with probucol molecules and coated with platelet membranes, thereby constructing a biomimetic multifunctional nanoplatform (BM-NP) with enhanced biological functionality. BM-NPs can selectively accumulate at AP-associated vascular injury sites through platelet membrane-mediated targeting. Under near-infrared (NIR) laser irradiation, the photothermal effect generated by PEG-MPDA facilitates the triggered release of probucol. PEG-MPDA synergizes with probucol to exert antioxidative effects, effectively scavenging ROS and reducing ox-LDL levels, ultimately inhibiting macrophage-derived foam cell formation. In vivo experiments demonstrated the targeted accumulation of BM-NPs within plaques. During PEG-MPDA treatment, BM-NPs exposed to NIR laser irradiation significantly reduced plaque deposition area, attenuated arterial intimal thickening, and lowered triglyceride (TG) levels in vivo. This engineered platelet membrane-based biomimetic nanoplatform (BM-NP) represents a potential nanotherapeutic strategy for precise and synergistic amelioration of AS [44]. Zhou et al. developed a biomimetic nanosystem, RAP@PLT NPs, by encapsulating rapamycin (RAP)-loaded PLGA NPs with platelet membranes. This system was combined with ultrasound-targeted microbubble destruction (UTMD) to facilitate drug release for AS treatment. The platelet membrane coating enhanced the targeting capability of RAP@PLT NPs toward foam cells, enabling localized RAP release at plaque sites. In an ApoE^−/−^ mouse model, UTMD-assisted RAP@PLT NPs significantly reduced the population of damaged smooth muscle cells (SMCs) and inflammatory macrophages, decreased lipid deposition and collagen content, markedly diminished plaque area, and improved plaque stability, thereby effectively inhibiting plaque progression. In summary, this study established a novel strategy for targeted nanoparticle delivery to APs by integrating the advantages of ultrasonic cavitation effects and biomimetic NPs in drug delivery [45]. Zhang et al. developed a biomimetic nanodelivery platform named PBP@siR@PM by coating black phosphorus nanosheets (BPNSs) loaded with small interfering RNA (siRNA) targeting Ca²⁺/calmodulin-dependent protein kinase gamma (CaMKIIγ) with platelet membranes. This platform enables targeted siRNA delivery. Leveraging platelet membrane coating, PBP@siR@PM specifically targets macrophages in APs, releases siRNA to suppress CaMKIIγ expression, and upregulates the cytoplasmic receptor MerTK. This restores macrophage efferocytosis, thereby enhancing the clearance of apoptotic cells within plaques. Additionally, PBP@siR@PM effectively scavenges ROS in macrophages, mitigates OS, and improves plaque stability. In atherosclerotic mouse models, this platform reduces cholesterol deposition and inflammation while significantly promoting macrophage efferocytosis, effectively inhibiting disease progression. The study demonstrates that platelet membrane-coated biomimetic NPs exhibit plaque-targeting capability, prolonged circulation half-life, enhanced biocompatibility, and protection of siRNA from premature degradation. This approach improves siRNA-mediated gene silencing efficacy and translational potential in diseased macrophages, highlighting its therapeutic promise for chronic inflammatory diseases like AS [46].

In summary, platelet membrane-coated NPs demonstrate significant advantages in the treatment of AS, exhibiting superior performance in targeted drug delivery. Utilizing natural platelet membranes to encapsulate NPs enables the carriage of various therapeutic agents, optimizes drug distribution and metabolism, avoids clearance by the immune system, prolongs circulation time in vivo, and efficiently targets lesion sites. However, this technology still faces numerous challenges, such as the complexity of the platelet membrane preparation process, which includes steps like platelet isolation, membrane extraction, and nanoparticle loading, hindering the large-scale application and promotion of platelet membrane-coated NPs. Additionally, the stability of platelet membranes both in vitro and in vivo may be compromised.

### Macrophage-derived extracellular vesicles

Extracellular vesicles (EVs) are naturally occurring nanosized vesicles enclosed by a phospholipid bilayer, measuring 40–100 nm in diameter. They serve as crucial mediators of intercellular communication and are widely distributed in bodily fluids. EVs contain various bioactive molecules and can transport diverse cargo, such as cytoplasmic proteins, membrane proteins, cytokines, chemokines, cell receptors, non-coding RNAs, mRNAs, and miRNAs. In AS lesions, EVs derived from endothelial cells, smooth muscle cells, and macrophages directly participate in plaque formation and progression by modulating processes such as inflammatory responses, reverse cholesterol transport, and vascular calcification. Macrophage-derived microvesicles (macrophage-EVs) possess inherent targeting capabilities and potential for immune evasion. Some nano-systems leverage “camouflage” strategies using macrophage vesicle membranes to achieve prolonged circulation in vivo and specific recognition of atherosclerotic lesion sites. This enables highly efficient targeted drug delivery for AS treatment, demonstrating broad prospects for therapeutic applications [47].

For example, Liang et al. collected macrophage-EVs secreted by RAW 264.7 cells via ultracentrifugation, loaded them with small interfering RNA targeting high mobility group box 1 (siHMGB1) using electroporation, and prepared macrophage-EV/siHMGB1 complexes. The results showed that the obtained macrophage-EV/siHMGB1 was effectively taken up by macrophages and inhibited the release of HMGB1 from macrophages. Further studies revealed that macrophage-EV/siHMGB1 could suppress macrophage polarization toward the M1 phenotype while promoting polarization toward the M2 phenotype. It also inhibited the uptake of ox-LDL by macrophages, thereby reducing foam cell formation. In an AS mouse model established using ApoE⁻/⁻ mice fed a high-fat diet, injection of macrophage-EV/siHMGB1 was found to inhibit macrophage infiltration into atherosclerotic plaque tissues, significantly reduce atherosclerotic plaques, and lower serum levels of TNF-α and IL-6. Mechanistically, macrophage-EV/siHMGB1 inhibited Caspase-11 activation, thereby suppressing macrophage pyroptosis and preventing the formation of atherosclerotic plaques. In summary, Liang et al. developed a macrophage-EV-based drug delivery platform. Macrophage-EV/siHMGB1 can inhibit the formation of AS plaques and suppress macrophage pyroptosis, thereby alleviating AS. Thus, macrophage-derived extracellular vesicles, as intelligent carriers for drug delivery, hold great potential for the clinical treatment of AS [48].

## Photo energy-derived nanotherapeutic strategy targeting macrophages

In recent years, with the deepening integration of nanotechnology and medicine, an increasing number of researchers have applied nanomaterials with unique photo-based energy to photothermal therapy (PTT) and photodynamic therapy (PDT) for the treatment of AS. These novel approaches have brought new hope for the treatment of AS (Fig. 1).

### Photodynamic therapy (PDT)

PDT is a novel non-invasive treatment method that utilizes light of specific wavelengths to activate photosensitizers. By modifying targeting ligands, PDT can precisely target macrophages, interfere with their activities, and thereby reduce inflammation within plaques [49]. PDT consists of three key components: photosensitizers, light, and oxygen molecules. Under appropriate excitation light, photosensitizers generate ROS such as singlet oxygen and peroxides, which subsequently induce a series of cytotoxic events. These include loss of mitochondrial membrane potential, lipid peroxidation, and protein denaturation in cell membranes and organelles [50]. Notably, high-intensity and low-intensity light irradiation can respectively trigger non-programmed cell death (necrosis) and programmed cell death (apoptosis and autophagy). The specificity of PDT, particularly for cardiovascular diseases like AS, depends on the precise localization and accumulation of photosensitizers within pathological target tissues [51, 52]. This specificity minimizes damage to surrounding healthy tissues. The efficacy of PDT in preclinical studies, including the reduction of intraplaque macrophage density and enhancement of plaque stability, depends on the specificity of the photosensitizer, optimal light dosage, and the effective targeting of photosensitizers within vascular structures. Recent exploration of NPs to improve photosensitizer delivery and specificity represents innovative progress in atherosclerotic PDT treatment. This advancement highlights the potential to enhance therapeutic efficacy and precision while reducing systemic toxicity (Table 2).

**Table 2:** Photo energy-derived nanotherapeutic strategy targeting macrophages

	Nanoparticles	Photosensitizer	Ligands	Targeting receptor	Model	Therapeutic effects	References
Photodynamic therapy (PDT)	CD68-Ce6-lip	Chlorin e6 (Ce6)	CD68	foam cells	Raw264.7-derived foam cells model	Promote autophagy and cholesterol efflux in foam cells; promote ROS generation in foam cells	[56]
	CS-CNCs@Ce6/DS	Ce6	dextran sulfate (DS)	macrophage	ApoE^–/–^ mice fed a high-fat diet	Promote ROS generation in macrophage; significant reduce the lesion area; inhibited cellular endocytosis of ox-LDL	[58]
	UCNPs-Ce6@DB	Ce6	dextran (DEX)	macrophage	ApoE^–/–^ mice fed a high-fat diet	Promote ROS generation and autophagy in macrophage; enhance cholesterol efflux; inhibit plaque formation	[59]
Photothermal therapy (PTT)	CuCo_2_S_4_	CuCo_2_S_4_	——	macrophage	ApoE^–/–^ mice fed a high-fat diet	Effectively ablate macrophages and reduce chronic inflammation of the carotid artery	[63]
	CoS_1.097_	Co	——	macrophage	Arterial inflammation and stenosis models of ApoE^–/–^ mice	Effectively suppress the thickening of the arterial wall by ablating inflammatory macrophages in the arterial wall; effectively inhibit the thickening of the intima/media of the arterial wall;	[64]
PTT + PDT	bTiO_2_-HA-p	bTiO_2_	hyaluronic acid (HA)	macrophage	ox-LDL induce RAW 264.7 cells to form foam cell model	Attenuate as high as 12.8% of intracellular lipids’ accumulation and the formation of macrophage-derived foam cells; activate SREBP2/LDLR pathway to reduce excess cholesterol uptake; promote ABCA1-dependent cholesterol efflux.	[65]
	DC/UCNPs@SiO_2_-RB/MB/CD36	Bengal red (RB)/methylene blue (MB)/ CeO_2_	CD36	macrophage	ApoE^–/–^ mice fed a high-fat diet	Significant reduce plaques area, the proportions of plaques in the aortic arch root and entire aortic arch were only 9.65% and 3.11% separately	[66]
Chemotherapy + PTT	MMS/Au/PTX/ VEGF/aV	Au NPs	vascular endothelial growth factor (VEGF)	Plaque vascular endothelial cells	ApoE^–/–^ mice fed a high-fat diet	Promote photothermal ablation of inflammatory macrophages; reduce the aortic lesion area by 18.2%	[67]
Sonodynamic therapy (SDT) + PTT	HA-HNSs	CuS/TiO_2_	HA	macrophage	ApoE^–/–^ mice perform partial carotid ligation surgery	Induce macrophage apoptosis; significant reduce the lesion area by 52.24%; reduce inflammatory factors	[68]

The first-generation photosensitizers are primarily based on the porphyrin macrocyclic structure of hematoporphyrin. These photosensitizers exhibit distinct selective accumulation properties when targeting APs. The ROS generated by these agents under light irradiation can disrupt macrophages, thereby inhibiting the progression of APs [53]. However, their clinical application has been limited by drawbacks such as relatively weak photodynamic efficacy.

Among the second-generation photosensitizers, derivatives of phthalocyanines, chlorin e6 (Ce6), purpurins, and benzoporphyrins (such as verteporfin) are considered promising for the treatment of AS. Second-generation photosensitizers offer advantages such as higher singlet oxygen formation rates and deeper tissue penetration due to their maximum absorption capacity in the 650–800 nm wavelength range [54]. Additionally, their higher tissue selectivity and rapid in vivo metabolism reduce the risk of systemic side effects. For instance, Kim et al. developed macrophage-targeted theranostic NPs (MacTNP) by conjugating the photosensitizer chlorin e6 (Ce6) with hyaluronic acid. This nanoformulation achieved active targeting of macrophages for photodynamic therapy. Macrophages highly express CD44 receptors on their surface, to which hyaluronic acid can bind, enabling active targeting. After lipopolysaccharide-activated macrophages internalized MacTNP, excessive peroxynitrite cleaved the chemical bonds in hyaluronic acid, triggering the release of the photosensitizer. Results demonstrated that under NIR irradiation, 66% of macrophages underwent necrosis, indicating that photodynamic therapy could significantly inhibit macrophage proliferation, thereby achieving therapeutic effects against AS [55]. Zou et al. developed a Ce6-loaded liposome using the membrane dispersion method and conjugated CD68 antibodies to the liposomal surface through covalent cross-linking reactions, constructing a CD68-modified Ce6-loaded liposome nanosystem (CD68-Ce6-lip). This system enables CD68-Ce6-lip to specifically target foam cells overexpressing CD68 receptors, thereby achieving therapeutic effects against AS. The results demonstrated that CD68-Ce6-lip promoted autophagy in foam cells by upregulating LC3-I and LC3-II expression while downregulating p62 expression. Furthermore, these NPs inhibited the migration of mouse aortic vascular smooth muscle cells (MOVAS) under laser irradiation and enhanced cholesterol efflux from foam cells, highlighting their potential as nanocarriers for photodynamic therapy of AS [56]. However, despite the many advantages demonstrated by these new photosensitizers, several challenges persist. For instance, photosensitizers such as phthalocyanines still suffer from drawbacks like slow clearance rates and poor water solubility, which limit their practical application in clinical treatments. Additionally, early-generation PDT drugs are activated at a wavelength of 700 nm. However, at this wavelength, the light delivery to target cells is significantly reduced by blood and tissues, thereby compromising the effectiveness of PDT [57].

The development of third-generation photosensitizers aims to enhance tissue targeting while improving the specificity and efficacy of PDT. Photosensitizers can be loaded into carriers such as micelles, polymer NPs, and self-assembling protein nanostructures to form nanocomposite materials. The integration of photosensitizers with nanomaterials can significantly boost the efficiency of photodynamic therapy. Photosensitizers can be encapsulated into nanocarriers through covalent or non-covalent interactions. Most photosensitizers are highly hydrophobic compounds that tend to aggregate in aqueous solutions, which not only reduces cellular uptake but also amplifies photothermal effects. For example, Liu et al. utilized a photothermal agent—carbon nanocages (CNCs)—to load the photosensitizer Ce6. Through electrostatic adsorption, DS was conjugated to the outermost layer, constructing a multifunctional nanoplatform named CS-CNCs@Ce6/DS, which achieved synergistic therapy combining PDT and PTT for AS. The DS component of the nanoplatform specifically binds to scavenger receptor A (SR-A) expressed on activated macrophages within APs, enabling targeted accumulation in the lesions. By applying sequential continuous laser irradiation at 808 nm and 603 nm, the photothermal effect enhanced PDT efficiency, leading to the ablation of activated macrophages. This process reduced the secretion of pro-inflammatory cytokines, alleviated the proliferation and migration of smooth muscle cells, and ultimately diminished AP burden. This approach represents a highly promising strategy for AS treatment [58]. Huang et al. developed a conjugate (DB) composed of dextran (DEX) and bovine serum albumin (BSA). Using DB as an emulsifier, they prepared upconversion NPs (UCNPs) and chlorin e6 (Ce6)-loaded nanoemulsions (UCNPs-Ce6@DB). The DEX modified on the nanoemulsion surface specifically recognizes and binds to scavenger receptor class A (SR-A), which is highly expressed on macrophages. This enables SR-A-mediated endocytosis to enhance the uptake of the nanoparticles by macrophage-derived foam cells within APs. Furthermore, UCNPs-Ce6@DB-mediated PDT amplifies ROS generation in macrophage-derived foam cells, induces autophagy, upregulates the expression of ABCA1 (a protein critical for cholesterol efflux), and suppresses the secretion of pro-inflammatory cytokines. In an AS mouse model, UCNPs-Ce6@DB was demonstrated to effectively inhibit plaque formation. These innovative therapeutic strategies exhibit significant potential in the field of AP management [59].

### Photothermal therapy (PTT)

PTT refers to a treatment modality that employs photothermal agents to absorb NIR and convert it into heat, thereby eliminating target cells. The efficacy of PTT relies on the ability of photothermal agents to generate localized hyperthermia in diseased areas upon irradiation with NIR light [60, 61]. This hyperthermia induces protein denaturation and disrupts cellular structures, ultimately leading to cell death [62]. Therefore, developing nanoplatforms based on photothermal therapy to ablate macrophages represents a feasible strategy for treating AS (Table 2).

Zhang et al. synthesized CuCo₂S₄ nanocrystals for PTT of AS. The CuCo₂S₄ nanocrystals exhibited excellent photothermal conversion capability. Experimental results demonstrated that CuCo₂S₄ combined with 808 nm NIR laser irradiation effectively ablated inflammatory macrophages. Furthermore, in ApoE^−/−^ mouse model, local injection of CuCo₂S₄ followed by 808 nm NIR laser irradiation significantly eliminated infiltrated inflammatory macrophages, markedly alleviating arterial inflammation and stenosis. These findings offer a novel strategy for PTT in AS treatment [63]. Lu et al. designed and synthesized CoS₁.₀₉₇ nanocrystals via a hydrothermal method as a novel nanoplatform for PTT of arterial inflammation. The CoS₁.₀₉₇ nanocrystals exhibited strong absorption in the NIR region, endowing them with exceptional photothermal performance. Notably, these nanocrystals possess degradation-triggered release capabilities for cobalt ions, which suppress macrophage proliferation caused by the slow release of cobalt ions. In an ApoE^−/−^ mouse model, under 808 nm laser irradiation, the CoS₁.₀₉₇ nanocrystals effectively ablated inflammatory macrophages on the arterial wall while inhibiting macrophage infiltration into the inflamed arterial tissue. This dual action significantly reduced arterial wall thickening and suppressed the progression of arterial stenosis. Consequently, CoS₁.₀₉₇ nanocrystals demonstrate substantial potential for PTT in AS [64].

### Photo energy-based synergistic therapy

Photo energy-based synergistic therapeutic approaches have demonstrated significant potential in the treatment of AS. Notably, the combination of PDT and PTT exhibits a strong synergistic effect, offering enhanced clinical translation for atherosclerotic management (Table 2). For instance, Dai et al. engineered porphyrin- and HA-modified black titanium dioxide (bTiO₂) NPs to achieve targeted synergistic PDT/PTT for foam cells. The results revealed that PDT alone induced substantial cell apoptosis or necrosis while only marginally reducing lipid levels. In contrast, the combined PDT/PTT modality activated the SREBP2/LDLR pathway to suppress intracellular cholesterol biosynthesis and external cholesterol uptake, while simultaneously enhancing ABCA1-mediated cholesterol efflux. This dual mechanism significantly reduced lipid accumulation in foam cells without triggering notable apoptosis or necrosis [65]. Gao et al. designed a multifunctional nanoplatform with dual up/downconversion emission (DC/UCNPs@SiO₂-RB/MB/CD36), functionalized with CD36 antibodies to enable targeted delivery to foam macrophages. Under 808 nm laser excitation, this nanoplatform demonstrated outstanding photothermal performance, enabling simultaneous in vivo optical imaging and PTT. Additionally, under 980 nm laser irradiation, the loaded photosensitizers (RB/MB) absorbed light to generate singlet oxygen (¹O₂), achieving PDT for AS. Crucially, the CeO₂ component catalyzed H₂O₂ in hypoxic microenvironments to enable in situ oxygen generation, further enhancing PDT efficacy. In ApoE^−/−^ mouse models, intravenous administration of DC/UCNPs@SiO₂-RB/MB/CD36 combined with NIR irradiation significantly cleared plaques at the aortic root and suppressed the secretion of pro-inflammatory cytokines (IL-1β and TNF-α). These results validate the nanoplatform’s ability to achieve synergistic PDT/PTT alongside NIR-II imaging for early-stage AS management [66].

Furthermore, the integration of chemotherapy with PTT has emerged as a common strategy for AS treatment (Table 2). For example, Li et al. developed a NIR-driven multifunctional micromotor by growing gold NPs (Au NPs) in situ on meso-/macroporous silica (MMS) and co-loading vascular endothelial growth factor (VEGF) with the anti-proliferative drug paclitaxel (PTX). This micromotor rapidly targets vascular cell adhesion molecule-1 (VCAM-1) on endothelial cells at atherosclerotic lesions. Its autonomous motion enhances penetration into plaque regions, while the photothermal effect under NIR irradiation enables efficient ablation of inflammatory macrophages. Additionally, the micromotor achieves rapid release of VEGF to promote endothelial repair and controlled release of PTX to inhibit smooth muscle cell proliferation, realizing a synergistic combination of photothermal and chemotherapy. In an AS mouse model, intravenous injection of the micromotor combined with NIR laser irradiation significantly suppressed lipid plaque formation. This drug-loaded micro/nanotechnology with hierarchical porous structures opens a promising therapeutic avenue for AS [67].

**Fig. 2:**
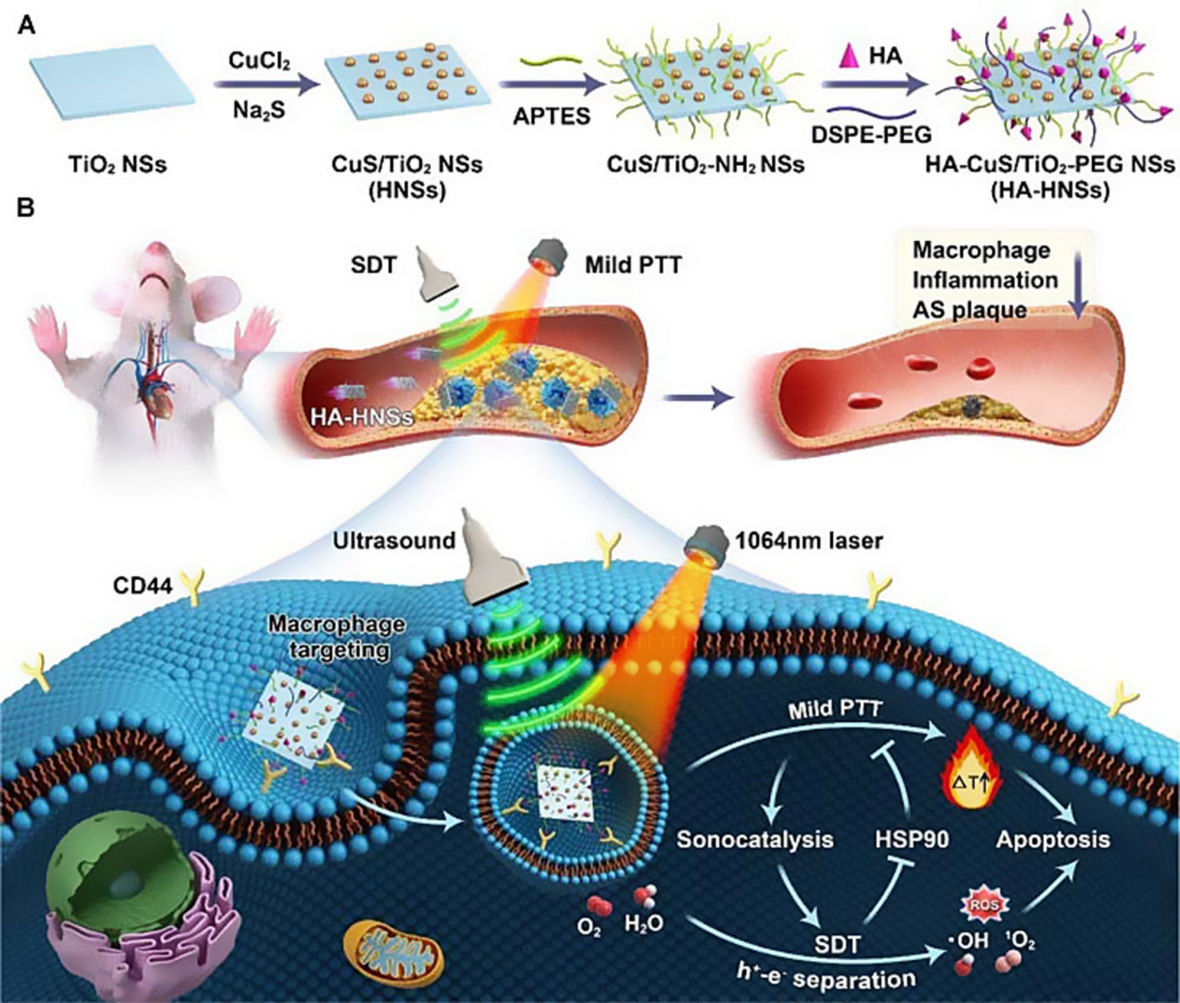
A combined therapy of photothermal therapy (PTT) and sonodynamic therapy (SDT). (**A**) Synthesis procedure of HA-HNSs and (**B**) HA-HNSs-mediated sonodynamic/photothermal synergistic therapy for preventing atherosclerotic plaque progression. Adapted with permission from [68], copyright 2022, American Chemical Society

The combination of sonodynamic therapy (SDT) and PTT represents an innovative approach for AS treatment (Table 2). SDT is a non-invasive, deep tissue-penetrating, and highly specific therapeutic modality. Upon activation by localized ultrasound (US), sonosensitizers generate ROS to induce apoptosis. The synergistic integration of SDT and PTT significantly enhances therapeutic efficacy against AS. For example, Cao et al. reported HA- and polyethylene glycol (PEG)-modified CuS/TiO₂ heterostructured nanosheets (HA-HNSs) for synergistic SDT and PTT in early-stage AP treatment. Due to their high surface-energy crystal facets and narrow bandgap, the heterostructured nanosheets (HNSs) exhibited exceptional sonodynamic performance. Simultaneously, HNSs demonstrated superior photothermal properties in the NIR-II region. In vitro experiments revealed that the photothermal effect enhanced the HA-HNSs-mediated sonocatalytic process, resulting in higher ROS generation. Furthermore, SDT-induced cleavage of HSP90 by abundant ROS synergistically improved the efficacy of mild PTT. HA modification enabled HA-HNSs to selectively target pro-inflammatory macrophages within plaques via CD44-HA interactions. The combined SDT/PTT approach synergistically induced apoptosis in these macrophages. In a mouse model of early-stage carotid AS, the dual therapy significantly suppressed plaque progression by ablating lesion-associated macrophages and reducing inflammation. HA-HNSs represent a safe and efficient sonodynamic/photothermal agent, offering substantial clinical translation potential for macrophage-targeted therapy in early AS (Fig. 2) [68].

Photo energy-based nanotherapy, as an innovative treatment approach, demonstrates significant potential in the field of atherosclerotic plaque therapy. Although notable progress has been made in optical nanotherapy, it still faces a series of challenges. First, the selectivity and targeting of phototherapy need to be improved to ensure that the treatment acts only on the affected areas, minimizing damage to surrounding healthy tissues. Second, further enhancement of light absorption and penetration depth is necessary to enable the application of optical nanotherapy to deeper-seated plaques. As research on photosensitizers continues to advance and technology evolves, we believe that photo energy-based nanotherapy will become one of the important methods for treating AS in the future.

## Macrophage-based microenvironment-responsive therapeutic strategies

The inflammatory microenvironment within plaques serves as a critical factor perpetuating chronic inflammation and driving the progression of AS. Macrophages play a pivotal role in mediating inflammatory regulation. Consequently, macrophage-centric modulation of the plaque microenvironment has emerged as a novel therapeutic target for AS, with strategies aimed at neutralizing or eliminating these inflammatory features within plaques gaining traction as innovative treatment paradigms. Developing stimuli-responsive NPs tailored to the pathological hallmarks of plaques—such as elevated ROS and low pH—enables precisely targeted therapy and controlled drug release at the disease site (Fig. 1). This approach enhances therapeutic efficacy while minimizing systemic side effects.

### ROS-based nanoplatform

ROS plays a significant role in the initiation and progression of AS by impairing endothelial function and promoting plaque formation. In AS, ROS may accumulate due to sustained inflammatory activity in local affected areas. Modulating the production of ROS is considered one of the effective therapeutic approaches. Therefore, it is crucial to regulate the progression of AS by clearing ROS in plaque macrophages, alleviating OS damage, and modulating macrophage polarization in the microenvironment.

#### ROS-responsive nanosystem

ROS-responsive nanomedicines have been developed to release drugs at target sites with excessive ROS production. Consequently, these NPs can both enhance therapeutic efficiency and reduce adverse effects (Table 3). Given the significantly elevated ROS levels in atherosclerotic microenvironments, numerous ROS-sensitive materials have been explored, including polythioether acetal, thioketal, ferrocene, and selenium-containing copolymers. These materials incorporate ROS-sensitive chemical bonds such as thioketal, thioether, or selenium-containing linkages, which cleave under ROS stimulation to release anti-inflammatory or antioxidant medications.

**Table 3:** Microenvironment-responsive therapeutic strategies for the treatment of atherosclerosis

	Nanoparticles	Carriers	Functional drug	Models	Therapeutic effects	References
ROS-Responsive	HASF@Cur micelles	oligomeric hyaluronic acid-2’-[propane-2,2-diyllbls (thio)] diacetic acl-hydroxymethylferrocene (oHA-TKL-Fc)	curcumin (Cur)	Rats fed a high-fat diet	Reduce ROS; reduce the lesion area of the aorta by 38%	[69]
	LLC NPs	heparin-lipoic acid conjugate (LMWH-LA)	Cur	ApoE^−/−^ mice fed a high-fat diet	Decrease ROS and inflammatory factors’ production; significantly reduce the luminal stenosis rate and collagen content in the plaque	[70]
	TPTS/C/T	α-tocopherol polyethylene glycol derivatives	Simvastatin (SIM)/ ticagrelor	ApoE^−/−^ mice fed a high-fat diet	Reduce ROS and inflammatory factors; reduce the plaque area by 63%	[71]
ROS-Scavenging	TN-PdHs	Palladium nanozyme	CAT-like	ApoE^−/−^ mice fed a high-fat diet	Reduce ROS and inflammatory factors; promote autophagy in macrophages; the plaque area reduce to 5.32%	[75]
	37pA-PtLNP Plus 37pA-LNP/6,877,002	Pt nanozymes	CAT-like/ TRAF6 inhibitor 6,877,002	ApoE^−/−^ mice fed a high-cholesterol diet	Reduce ROS and inflammatory factors; 65.7% and 83.0% reductions in the ratio of aortic plaque area and abdominal aortic plaque area, respectively;	[76]
	HCN@DS	hollow carbon nanospheres (HCNs)	POD-like, CAT-like, and OXD-like	ApoE^−/−^ mice fed a high-fat diet	Reduce ROS; reduce the plaque area by 19.18%; reduce the collagen content decrease by 25.3%; reduce the macrophage content by 20.7%	[77]
pH-Responsive	SIM/ZIF-8@HA	zeolitic imidazolate framework-8 (ZIF-8) NPs	SIM	ApoE^−/−^ mice fed a high-fat diet	Reduce ROS; reduce lipid deposited by 16.1%; significant reduction in atherosclerotic lesions, the lesion-to-aorta ratio is only 11.5%	[78]
	H-CuS@DMSN-N = C-HA	mesoporous silica NPs/ copper sulfide NPs	heparin (Hep)	FeCl_3_-induced carotid artery damage model of rabbits	Effectively reduce the formation of a thrombus	[79]
	ST/NCP-PEG	benzoic-imine (BI) linker and Gd^3+^	SIM	ApoE^−/−^ mice fed a high-fat diet	Reduce ROS; reduce the M1/M2 ratio by 85%	[80]
	BC@CS/cRGD	chondroitin sulfate (CS)	baicalin (BC)	ApoE^−/−^ mice fed a high-fat diet	Exhibit the greatest reduction in lipogenesis; reduce the number of neo-endothelial cells in aortic arch plaques; reduce the expression of MMP-9	[81]
	PIP-CD47	polydopamine	ibrutinib	ApoE^−/−^ mice fed a high-fat diet	Reduce ROS and inflammatory factors; reduce the lipid area by 26.99%; enhance plaque stability; effectively kill B lymphocytes in the arterial region	[82]

**Fig. 3:**
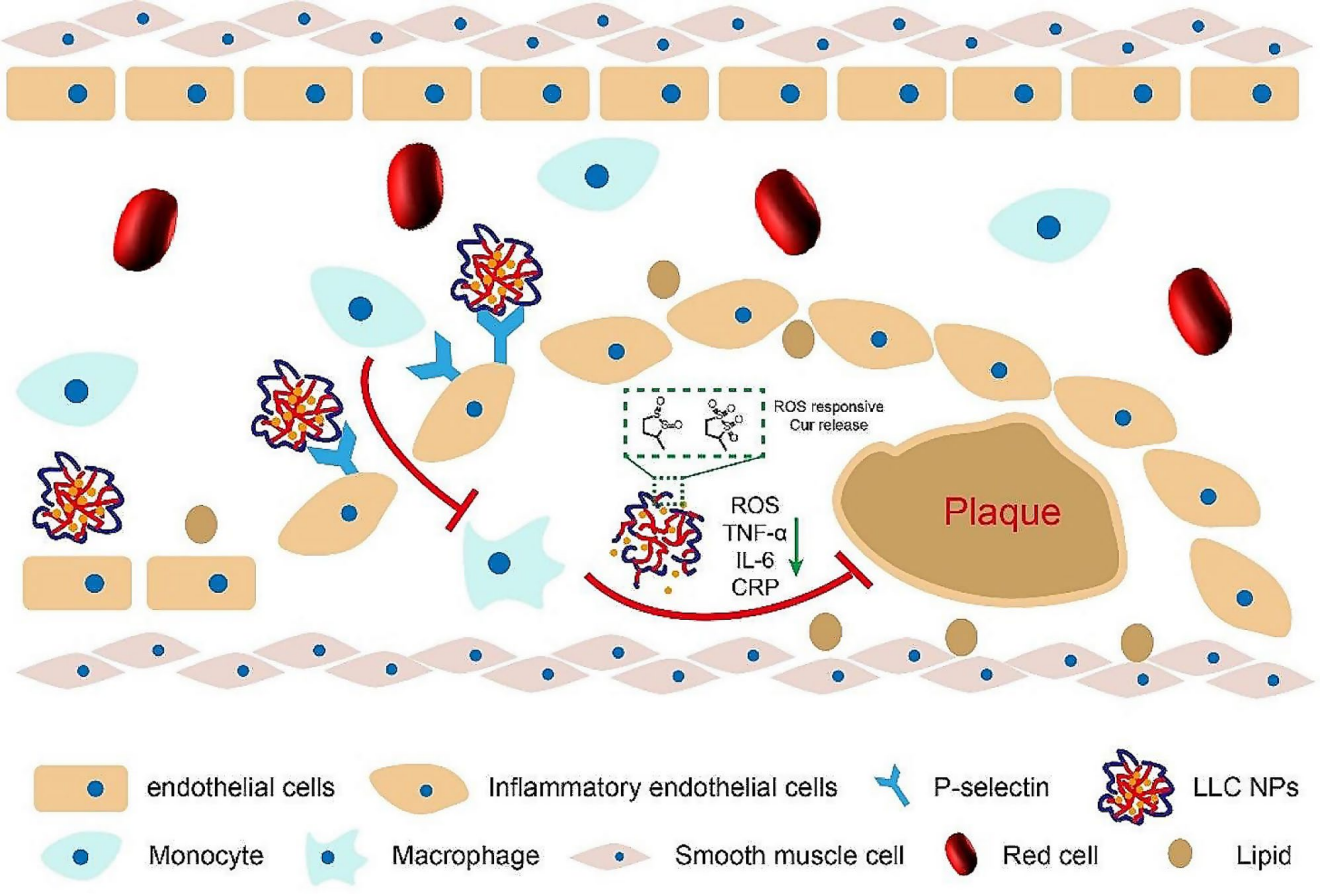
A nanotherapeutic cascade strategy to inhibit plaque inflammation for enhanced anti-atherosclerosis therapy. Adapted with permission from [70], copyright 2024, BioMed Central

For example, Hou et al. synthesized a novel ROS-sensitive and CD44 receptor dual-targeting amphiphilic carrier material, oligomeric hyaluronic acid-2’-[propane-2,2-diylbis(thio)]diacetic acid-hydroxymethyl ferrocene (oHA-TKL-Fc), and encapsulated curcumin (Cur) with HASF into nanomicelles (HASF@Cur micelles). Results demonstrated that HASF@Cur micelles exhibited ROS-responsive properties, where the thioketal linkages in HASF@Cur micules would cleave to release Cur under elevated ROS levels. In vivo studies revealed that after administration in mice, HASF@Cur micules could specifically target atherosclerotic plaques and significantly reduce aortic lesion areas. This ROS-sensitive and CD44 receptor dual-targeting drug delivery system represents a promising strategy for AS treatment [69]. Luo et al. employed an amphiphilic low-molecular-weight heparin-lipoic acid conjugate (LMWH-LA) as a ROS-sensitive carrier material to load curcumin (Cur), fabricating LLC NPs. The disulfide bonds in LA not only exhibited antioxidant activity but also demonstrated ROS-triggered hydrophilic-hydrophobic transition properties, enabling targeted Cur release within APs to exert anti-inflammatory and antioxidant effects. Low-molecular-weight heparin could specifically target P-selectin on plaque endothelial cells, competitively blocking monocyte migration to endothelial cells while suppressing macrophage recruitment, thereby reducing ROS and inflammatory factor production. In atherosclerotic mouse models, LLC NPs were found to significantly decrease macrophage content within plaques, reduce intraplaque lipid accumulation, lower luminal stenosis rates, and diminish collagen deposition. These findings indicate that LLC NPs exhibit remarkable therapeutic efficacy against AS (Fig. 3) [70].

Furthermore, targeted therapy can be achieved through ROS-responsive prodrug activation, enabling localized activation of the therapeutic agent at the disease site. This strategy involves covalent conjugation of ROS-cleavable linkages to hydroxyl groups on lactone rings. The prodrug remains stable and pharmacologically inert in circulation under physiological ROS levels in the bloodstream. However, when ROS concentrations elevate within APs, the ROS-responsive bonds undergo cleavage, releasing the prodrug which subsequently undergoes structural conversion to attain pharmacological activity through precise chemical transformation mechanisms. For instance, Zhao et al. synthesized a ROS-responsive simvastatin nanoprodrug (TPTS NPs) by conjugating α-tocopherol polyethylene glycol derivatives with the pharmacophore of HMG-CoA reductase inhibitor simvastatin via a thioketal linker. Subsequently, they functionalized TPTS NPs with targeting peptide CREKA, while simultaneously loading ticagrelor, to construct an advanced ROS-activated nanosystem (TPTS/C/T). TPTS/C/T can target AP sites through encapsulated CREKA peptides and achieve spatiotemporal co-delivery of simvastatin, α-tocopherol, and ticagrelor. Under high ROS levels, thio-simvastatin is released via cleavage. Subsequently, the ester bond adjacent to the sulfhydryl group undergoes hydrolysis, releasing the parent simvastatin, α-tocopherol, and ticagrelor. The findings demonstrated that TPTS/C/T significantly inhibited M1 polarization of macrophages and reduced intracellular ROS and inflammatory cytokine levels. In the ApoE^−/−^ mouse model, TPTS/C/T effectively targeted AP sites, releasing simvastatin and ticagrelor, which notably diminished the necrotic core area within plaques. This study provides novel insights into the design of ROS-responsive drug-releasing nanosystems for AS therapy [71].

#### Nanozymes for ROS scavenging

In recent years, nanomaterials with unique ROS-regulating properties have laid the foundation for a new generation of anti-atherosclerotic therapeutic approaches. Scavenging excessive ROS in AS, reducing OS, and decreasing the production of lipid peroxides can thereby reduce plaque formation. Certain endogenous enzymes, including superoxide dismutase (SOD), catalase (CAT), and glutathione peroxidase (GPx), possess the ability to eliminate ROS [72, 73]. O₂ is catalyzed by SOD to generate H₂O₂. Subsequently, CAT, GPx, and glutathione (GSH) can effectively convert H₂O₂ into non-toxic products, thereby preventing the accumulation of ROS. Due to the poor stability of natural enzymes and challenges in large-scale production, their applications are limited. Therefore, developing nanomaterials with enzyme-mimicking properties for ROS regulation is crucial. Certain nanomaterials exhibit antioxidant enzyme-like activities (e.g., SOD and CAT), enabling them to directly neutralize superoxide anions (O₂⁻), H₂O₂, and hydroxyl radicals (·OH). For instance, nanozymes can restore oxidative states to normal ROS levels by eliminating H₂O₂, thereby reducing oxidative damage. Nanozymes exhibit significant advantages in scavenging ROS. Compared to natural enzymes, they possess broad-spectrum ROS scavenging capabilities, demonstrate strong stability under physiological conditions, and exhibit excellent biocompatibility and biosafety. More importantly, nanozymes can be engineered to enhance their performance, enabling catalytic capabilities that surpass those of classical enzymes. Depending on their constituent materials, nanozymes can be categorized into various types including metal-based, non-metal-based, and metal-organic framework (MOF)-based nanozymes [74]. To date, numerous nanozymes have been developed and demonstrated therapeutic potential for treating APs (Table 3).

**Fig. 4:**
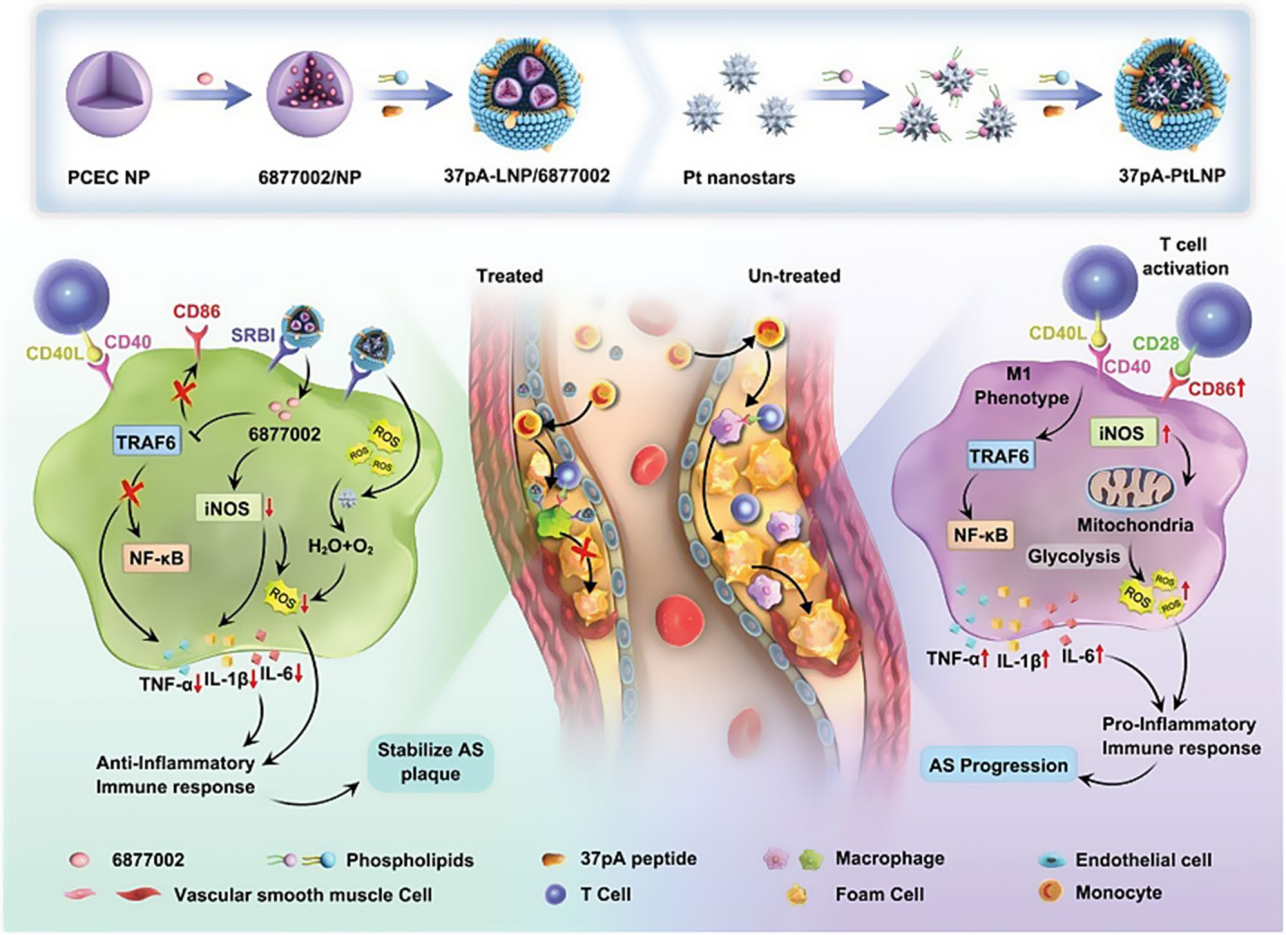
The nanosystems 37pA-LNP/6,877,002 and 37pA-PtLNP based on nanozymes regulate the plaque microenvironment. During the atherosclerosis therapy, 37pA-PtLNP can effectively scavenge intracellular ROS of inflammatory macrophage in plaque. On the other side, 37pA-LNP/6,877,002 can inhibit the activities of TRAF6 to modestly decrease the proportion of the proinflammatory macrophage phenotype (reduce the expression of CD86 and iNOS). More importantly, the combination of 37pA-PtLNP and 37pA-LNP/6,877,002 can regulate the atherosclerotic plaque microenvironment and achieve satisfactory stabilization of plaques with minimal progression. Adapted with permission from [76], copyright 2023, Wiley-VCH Verlag

For example, Hu et al. developed a unique palladium-based nanozyme with a tetra-pod cone-shaped spiny structure (TN-PdHs), which effectively treats AS through ROS scavenging, anti-inflammatory effects, and autophagy activation. Due to their excellent CAT-like activity, TN-PdHs can efficiently eliminate ·OH radicals and significantly reduce lipid peroxidation levels. Furthermore, the distinctive spiny morphology of TN-PdHs strongly induces autophagy responses in macrophages. Additionally, TN-PdHs exhibit potent anti-inflammatory effects by regulating various inflammation-related signaling pathways. Both in vitro and in vivo experimental results demonstrate the synergistic interplay among antioxidant, anti-inflammatory, and autophagy-activating properties of this multifunctional nanozyme, providing theoretical support for developing nanomedicines against AS [75]. Yang et al. constructed a 37pA-PtLNP nanozyme by modifying lipid-coated Pt nanozymes with 37pA peptide, and simultaneously developed 37pA-LNP/6,877,002 NPs by modifying lipid NPs loaded with TRAF6 inhibitor 6,877,002 using 37pA peptide. Through synergistic application of 37pA-PtLNP and 37pA-LNP/6,877,002, they developed a macrophage-targeted nanosystem. This nanosystem (37pA-PtLNP Plus 37pA-LNP/6877002) exhibited CAT-activity, effectively eliminating intracellular ROS including ·O2-, ·OH, and H2O2 radicals, thereby alleviating the inflammatory environment caused by excessive ROS production in plaques. The introduction of TRAF6 inhibitor 6,877,002 inhibited CD40-induced activation of the NF-κB pathway, consequently suppressing macrophage polarization to the M1 phenotype and reducing M1 macrophage-mediated production of inflammatory cytokines. In ApoE^−/−^ mouse models, administration of 37pA-PtLNP Plus 37pA-LNP/6,877,002 was shown to enhance mitigation of chronic inflammatory microenvironments by reducing oxidative stress and downregulating pro-inflammatory cytokines and chemokines expression, while significantly reducing aortic plaque area and abdominal aortic plaque area (Fig. 4) [76]. Wang et al. successfully constructed a novel metal-free nanozyme (HCN@DS) by loading DS onto the surface of hollow carbon nanospheres (HCNs), which targets macrophages to eliminate APs. HCN@DS exhibits POD-like, CAT-like, and oxidase-like (OXD-like) activities, enabling it to scavenge excessive ROS generated in APs. The resulting moderate ROS levels can further enhance autophagy in inflammatory macrophages. More importantly, under NIR-II irradiation, HCN@DS utilizes mild photothermal effects (< 45 °C) to activate autophagy in macrophages or foam cells. This process simultaneously inhibits foam cell formation through two mechanisms: reducing ox-LDL uptake and promoting macrophage efferocytosis, while activating signaling pathways that promote cholesterol efflux in macrophages. Consequently, this approach enhances plaque stability and suppresses the progression of AS [77].

### pH-responsive nanosystem

Atherosclerotic lesions are typically hypoxic, characterized by elevated lactate concentrations and localized acidification of the extracellular fluid. This acidification may result from lipid-laden macrophages within the lesions. The phagocytosis of ox-LDL by macrophages to form foam cells is an ATP-dependent process. Lipid overload forces macrophages to rely on anaerobic glycolysis for energy production, leading to lactate accumulation and a subsequent decline in the pH of the plaque microenvironment. In atherosclerotic lesions, the pH drops from approximately 6.8 to 5.5. Consequently, pH-responsive nanomedicines have been designed to modulate the inflammatory microenvironment for the treatment of AS (Table 3).

pH-responsive polymeric materials typically contain weakly acidic or basic groups, such as carboxylic or amine groups. These groups undergo protonation or deprotonation, enabling the capture or release of protons in response to changes in external pH. By leveraging the acidic microenvironment of APs, pH-responsive nanoplatforms have been developed to achieve effective treatment of AS.

**Fig. 5:**
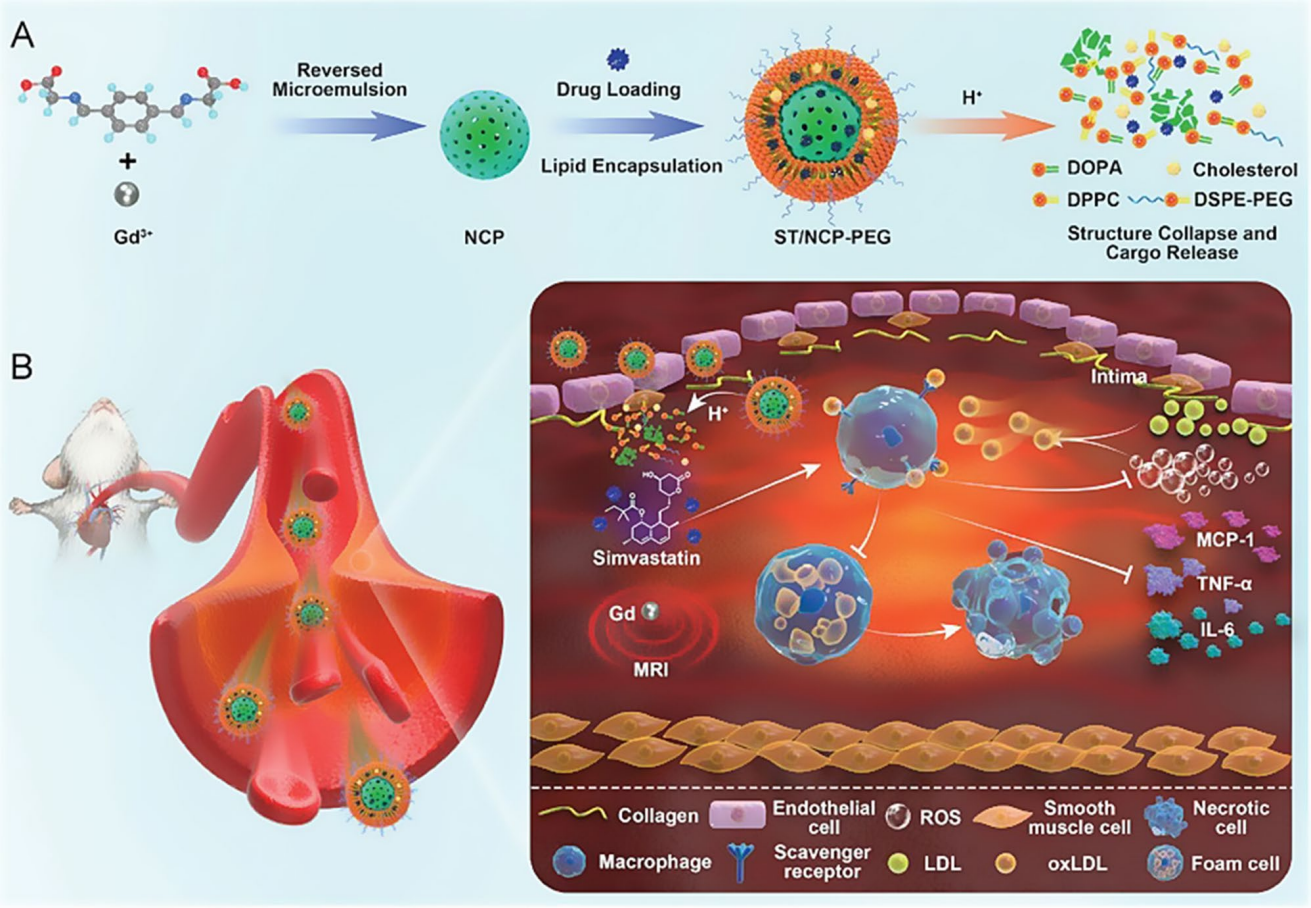
Design strategies for pH-responsive nanoplatforms. (**A**) the fabrication procedure of ST/NCP-PEG and pH-responsive structural collapse under acidic microenvironment. (**B**) the mechanism of pH-responsive and MRI-functional ST/NCP-PEG nanomedicine enabling spontaneous diagnosis and treatment of atherosclerosis. Adapted with permission from [80], copyright 2024, Wiley-VCH Verlag

For example, Obaid et al. developed HA-coated zeolitic imidazolate framework-8 (ZIF-8) NPs loaded with simvastatin (SIM), termed SIM/ZIF-8@HA nanoplatforms, for the treatment of AS. This nanoplatform efficiently accumulated in plaque regions through specific recognition between HA and CD44. Simultaneously, it decomposed in the acidic atherosclerotic microenvironment to release SIM. Results showed that under pH 5 conditions, 87.5% of SIM was released from SIM/ZIF-8 after 48 h of incubation due to the disruption of coordination bonds between zinc ions and 2-methylimidazole in ZIF-8 under acidic conditions. However, no significant SIM release was observed from the NPs when incubated in neutral solutions for 48 h. In vivo therapeutic studies in mice demonstrated that SIM/ZIF-8@HA effectively reduced plaque area, suppressed inflammatory infiltration, and stabilized vulnerable plaques [78]. Liu et al. developed a multifunctional nanosystem (H-CuS@DMSN-N = C-HA) using mesoporous silica NPs (MSNs) as carriers to deliver the anticoagulant drug heparin (Hep). The pores of the NPs were sealed with photothermal copper sulfide NPs (CuS NPs), and oxidized hyaluronic acid (oxi-HA) was attached to the NP surface via pH-sensitive Schiff base bonds. The HA coating endowed the nanocomposite with pH responsiveness and the ability to target CD44-positive inflammatory macrophages. Both in vitro and in vivo studies confirmed that this nanosystem could achieve photothermal ablation of macrophages and thrombi under NIR irradiation, while the pH-sensitive Schiff base bonds hydrolyzed in the weakly acidic microenvironment of atherosclerotic inflammation to release the drug. This approach realized precise drug delivery, controlled drug release, and chemo-photothermal synergistic therapy for AS [79]. Lin et al. synthesized pH-responsive nanoscale coordination polymers (NCPs) using gadolinium ion (Gd³⁺)-crosslinked benzoic-imine (BI) linkers. They then loaded SIM onto the surface of the NCPs to prepare ST/NCP-PEG NPs. The BI linkers in these NPs remain stable at pH 7.4 but readily hydrolyze in acidic environments. After intravenous injection, the ST/NCP-PEG NPs effectively accumulate within APs. Under the mildly acidic conditions of the plaques, the NPs degrade to release SIM, which reduces inflammation and OS while shifting macrophage phenotypes from pro-inflammatory M1 to anti-inflammatory M2. Additionally, ST/NCP-PEG enables spontaneous magnetic resonance imaging (MRI) of plaques through the release of Gd³⁺ complexes, allowing effective detection of atherosclerotic lesions. These ST/NCP-PEG NPs represent a promising theranostic strategy for autonomous diagnosis and treatment of AS (Fig. 5) [80]. Deng et al. prepared a pH-responsive nanosystem, BC@CS/cRGD NPs, by conjugating baicalin (BC) with chondroitin sulfate (CS) through an amidation reaction, followed by modification with the targeting fragment cRGD peptide. Due to the presence of amide bonds, BC@CS/cRGD NPs could rapidly hydrolyze and release BC under low pH conditions. In vitro release experiments demonstrated that the NPs released BC more rapidly at pH 5.0 compared to pH 7.4. The cRGD peptide enables BC@CS/cRGD NPs to simultaneously target both damaged endothelial cells and inflammatory macrophages by specifically binding to the αvβ3 protein receptor overexpressed in these cells. In a mouse AS model, BC@CS/cRGD NPs accumulated at plaque sites, where BC was released in the acidic microenvironment of plaques to restore endothelial function. Concurrently, it promoted macrophage polarization from the M1 to M2 phenotype, suppressed inflammatory responses, and effectively attenuated plaque progression. In summary, this pH-sensitive BC@CS/cRGD NPs offers a promising strategy for treating AS by modulating the inflammatory microenvironment [81]. Wang et al. developed a targeted nanodrug delivery system named PIP-CD47 by conjugating anti-CD47 antibodies to the surface of polydopamine NPs encapsulating ibrutinib. The synthesized nanodrug PIP-CD47 leverages CD47 monoclonal antibodies and pH-responsive release properties to achieve active targeting, enabling precise delivery of the Bruton’s tyrosine kinase (BTK) inhibitor ibrutinib to APs. The system utilizes pH-sensitive reversible dissociation of polydopamine to achieve controlled drug release in pathological mildly acidic microenvironments. PIP-CD47 exerts therapeutic effects by releasing ibrutinib to suppress NF-κB pathway activation in plaque B-cells and NLRP3 inflammasome activation in macrophages. This effectively reverses M1 macrophage polarization while reducing M1-associated pro-inflammatory factors, ROS, and nitric oxide (NO) levels. In atherosclerotic mouse models, PIP-CD47-mediated targeted inhibition of BTK signaling pathways in macrophages and B-cells at plaque sites significantly attenuated aortic lesion severity. The system also enhanced plaque stability, mitigated excessive inflammation, and modulated the immune cell microenvironment within plaques. This study proposes a novel strategy for targeted therapy development and drug design in AS treatment [82].

## Limitations in the clinical translation of nano-therapeutics for atherosclerosis

However, based on current research, the success of nanotherapies in preclinical studies does not guarantee their efficacy in humans. In animal models, AS is typically established within weeks, whereas the formation of atherosclerotic plaques in patients takes years or even decades, making the human condition more complex and heterogeneous. Most existing animal models used to study AS are primarily based on small mammalian species, such as the two classic transgenic mouse models ApoE⁻/⁻ and LDLR⁻/⁻, which exhibit inherent differences from humans [83]. To address the limitations of classical mouse models in replicating human-like lipoprotein metabolism, the ApoE*3-Leiden mouse was developed in the 1990s. In these mice, the clearance of non-HDL lipoproteins is impaired. Unlike ApoE⁻/⁻ mice, which completely lack ApoE, this model expresses detectable levels of endogenous ApoE. Compared to the non-physiological total cholesterol (TC) levels observed in ApoE⁻/⁻ and LDLR⁻/⁻ mice, ApoE*3-Leiden mice develop moderate hyperlipidemia and form well-defined lesions. When crossbred with human CETP transgenic mice, ApoE*3-Leiden.CETP (E3L.CETP) mice exhibit humanized lipoprotein metabolism, characterized by a shift from HDL to elevated VLDL/LDL fractions, reduced SR-B1-mediated cholesterol efflux, and severe lesion progression. Compared to ApoE⁻/⁻ and LDLR⁻/⁻ mice, the E3L.CETP model more closely mimics human lipoprotein metabolism, making it a preferred model for studying lipid metabolism and reverse cholesterol transport (RCT) pathways. Consequently, E3L.CETP mice may offer better translational relevance to humans. However, these models have not yet been utilized in preclinical studies of macrophage-targeted nanotherapies for AS [83]. Additionally, it is recommended to feed these model mice diets that only moderately increase plasma cholesterol levels and allow atherosclerosis to develop over an extended period, more closely resembling the progression of human AS.

This is particularly important given that macrophages are distributed across various tissues and organs, which necessitates the specific targeting of plaque macrophages by NPs. Therefore, future research should focus on ensuring that NPs are designed to specifically target plaque macrophages while minimizing non-specific uptake by macrophages in other tissues.

Furthermore, while existing studies have investigated the biosafety of nanomedicines in visceral organs such as the heart, liver, spleen, lungs, and kidneys, their potential impact on the nervous system remains unexplored. Another critical consideration is that although NPs have demonstrated safety and efficacy in other pathological contexts, they may exert unintended effects on AS. AS is driven by chronic inflammation in the vascular wall. Therefore, even if an NP formulation successfully localizes to plaques, it could exacerbate disease progression if the nanomaterials inadvertently stimulate inflammatory responses. Additionally, the systemic immune responses modulated by NPs must be thoroughly investigated before these strategies can be advanced to clinical trials.

## Conclusions and prospects

AS is a chronic inflammatory disease primarily affecting large- and medium-sized arteries, serving as the main pathological basis for cardiovascular diseases such as coronary heart disease. Although the widely adopted clinical strategy of lipid-lowering therapy has achieved favorable treatment outcomes, single-target lipid-lowering approaches cannot completely cure AS, and the toxic side effects of lipid-lowering drugs limit their clinical application [84]. With advancing understanding of AS mechanisms, numerous innovative nanotherapeutic systems for diagnosis and treatment have been developed. Compared with conventional drugs, nanomedicines offer the following advantages: First, surface functionalization of NPs with targeting ligands enables precise targeting of APs, minimizing off-target effects. Macrophage-targeted nanotherapeutic strategies can reduce plaque burden and inflammation through synergistic effects on apoptosis, autophagy, and efferocytosis induction. Second, bioinspired cell membrane-coated NPs retain inherent cellular characteristics and functions, demonstrating enhanced immune evasion capabilities while exerting anti-atherosclerotic effects through their unique immunomodulatory and targeting properties. Third, NPs can achieve intelligent stimuli-responsive drug release in inflammatory microenvironments, enabling site-specific clearance of atherosclerotic lesions. Finally, NPs can co-deliver multiple anti-atherosclerotic agents to achieve spatiotemporally precise combination therapy [85, 86]. Therefore, nanotechnology shows great promise in the diagnosis and treatment of AS.

However, the clinical translation of these nanomaterials also faces several challenges. For instance, the inherent atherosclerotic microenvironment (such as its acidity) may weaken the therapeutic effects of PTT and PDT. Therefore, it is essential to develop multifunctional nanosystems responsive to the AP microenvironment, which can synergistically exert therapeutic effects through multiple mechanisms, including the loading of chemical agents such as anti-inflammatory or cholesterol-lowering drugs. Additionally, certain nanomaterials possess unique enzymatic activities, such as nanozymes, which can eliminate ROS and lipid peroxidation. Consequently, creating novel ROS-responsive nanosystems by synergizing nanozymes could effectively treat AS and related vascular diseases [87, 88]. Furthermore, the atherosclerotic microenvironment contains abundant biological molecules such as proteins, lipids, and nutrients. These biomolecules can adsorb onto the surface of NPs, forming a layer termed the “biomolecule corona”. For example, the biomolecule corona may alter their biodistribution in vivo and modulate their interactions with the immune system [89]. Thus, it is critical to consider the interplay between NPs and biomolecules to understand their behavior in the atherosclerotic microenvironment. More importantly, most nanomedicines have only undergone phenotypic studies (e.g., evaluating anti-inflammatory and antioxidant effects, as well as their impact on cholesterol efflux), necessitating further investigation into their precise mechanisms, including the exact target sites and molecular interactions. The interactions between NPs and pathological tissues must be fully elucidated and validated in large animal models.

In conclusion, with the continuous elucidation of atherosclerotic pathogenesis and remarkable advancements in bionanotechnology, synergistic nanosystems integrating novel therapeutic strategies have facilitated new progress in AS treatment. An increasing number of nanomedicine candidates are expected to enter clinical trials, heralding a new era in the management of AS.

## Data Availability

Not applicable.
